# Supplementary material for: Effects of Processing on Starch Structure, Textural, and Digestive Property of “Horisenbada”, a Traditional Mongolian Food
Source: Foods. 2022 Jan 13;11(2):212. doi: 10.3390/foods11020212 (PMC8774302; doi:10.3390/foods11020212)
Supplement: Supplementary file 1 [file foods-11-00212-s001.zip › foods-1515991-supplementary-final version.pdf]

Effects of processing on starch structure, textural, and digestive property of "Horisenbada", a traditional mongolia food

Li Hongyan[1], Chen Zhijun[1], Yifan Mu[1], Ma Ruolan[1], Laxi Namujila[2,3], Fu Minghai[2,3*]

1 *China-Canada Joint Lab of Food Nutrition and Health (Beijing), School of Food and Health, Beijing Technology and Business University (BTBU), 11 Fucheng Road, Beijing 100048, China*

2 *NMPA Key Laboratory of Quality Control of Traditional Chinese Medicine (Mongolian Medicine), Inner Mongolia University for Nationalities, Tongliao 028000, China*

3 *School of Mongolian Medicine, Inner Mongolia University of Nationalities, Tongliao, Inner Mongolia 028000, China*

* Correspondence: mfu@imun.edu.cn

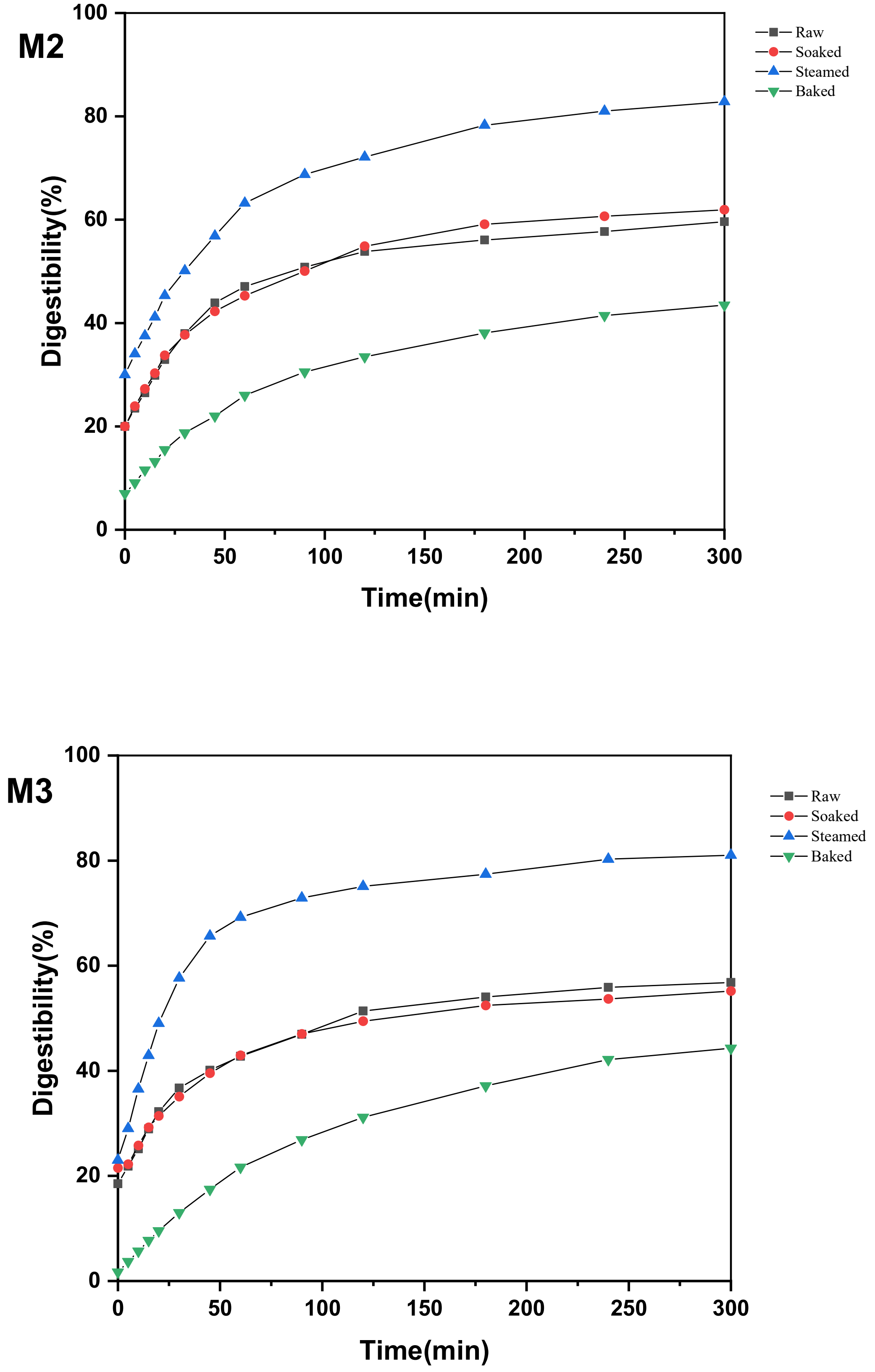

Fig. S1 The digestibility of raw and soaked, steamed, and baked M2 and M3 millets

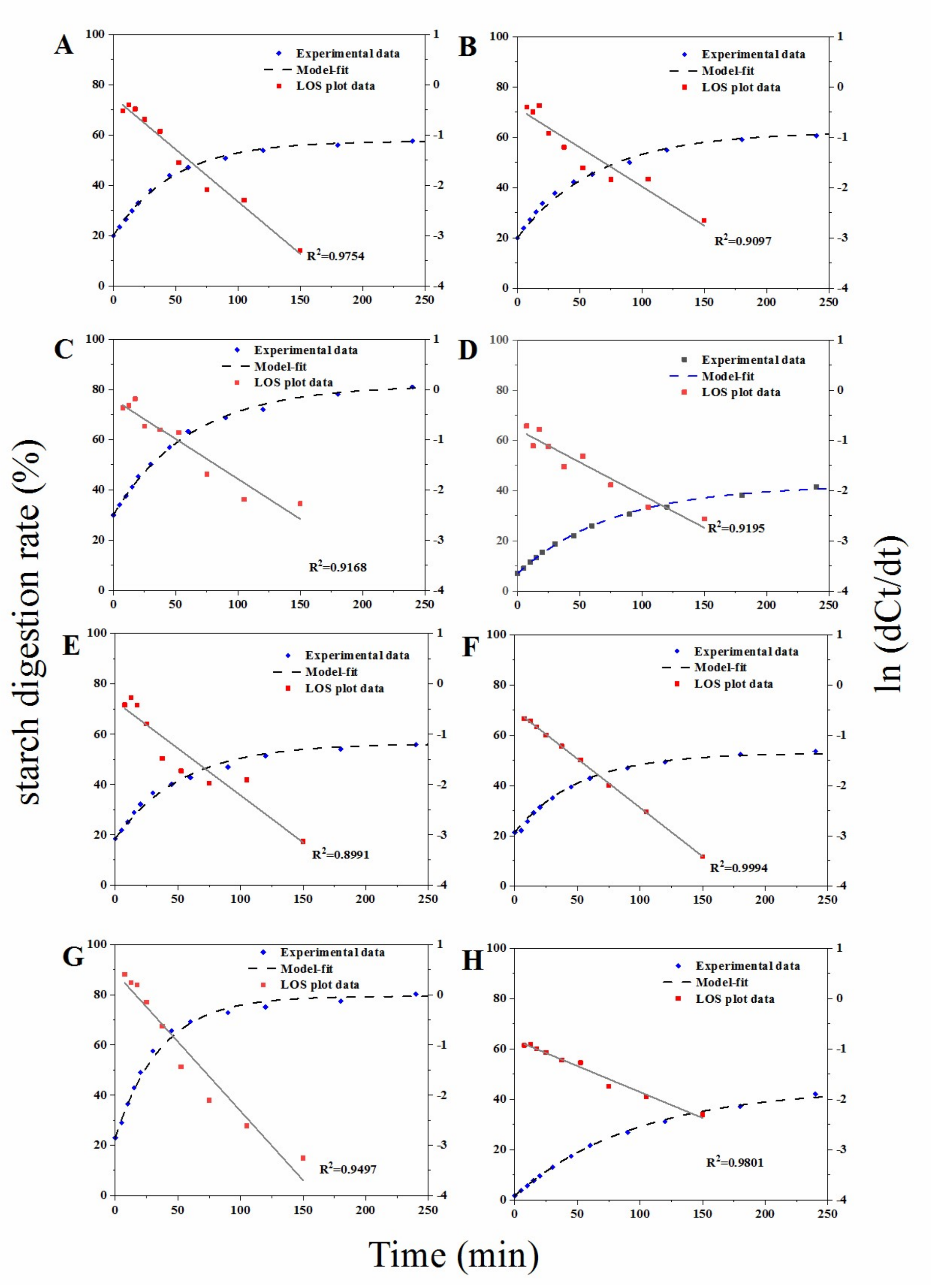

Fig. S2 Typical starch digestion curves, LOS plots and model-fit curves from M2 and M3 millets by different processes. A, B, C, D represent native millet, soaked, steamed, and baked millets of M2, respectively; E, F, G, H represent native millet, soaked, steamed, and baked millets of M3, respectively. The R-squared values relate to the LOS plots.
